# Supplementary material for: Physicians’ knowledge about palliative care in Bangladesh: A cross-sectional study using digital social media platforms
Source: PLoS One. 2021 Sep 1;16(9):e0256927. doi: 10.1371/journal.pone.0256927 (PMC8409647; doi:10.1371/journal.pone.0256927)
Supplement: S1 Appendix — (DOCX) [file pone.0256927.s001.docx]

1. **Socio-demographic information:**
2. **Age:**
3. **BMDC Reg No:**
4. **Educational Status**:
5. Graduate b. Postgraduate
6. **Gender:**
7. Male b. Female c. Others
8. **Specialty:**
9. Medicine b. Surgery c. Gynecology d. Pediatrics e. Basic medical sciences f. Public health g. General practitioner h. Intern i. Others (specify)

**5. Are you familiar with the term “Palliative Care”?**

a. Yes b. No

1. **Assessing knowledge level about palliative care (PaCKs)**

Below are some questions about a type of care called palliative care. Please answer the questions below by circling True, False, or I don’t know. If you do not know the answer, do not guess. Instead, respond with “I don’t know.”

**1. A goal of palliative care is to address any psychological issues brought up by serious illness.**

1.True 2. False 3. I don’t know

**2. Stress from serious illness can be addressed by palliative care.**

1.True 2. False 3. I don’t know

**3. Palliative care can help people manage the side effects of their medical treatments.**

1.True 2. False 3. I don’t know

**4. When people receive palliative care, they must give up their other doctors.**

1.True 2. False 3. I don’t know

**5. Palliative care is exclusively for people who are in the last 6 months of life.**

1.True 2. False 3. I don’t know

**6. Palliative care is specifically for people with cancer.**

1.True2. False 3. I don’t know

**7. People must be in the hospital to receive palliative care.**

1.True 2. False 3. I don’t know

**8. Palliative care is designed specifically for older adults.**

1.True 2. False 3. I don’t know

**9. Palliative care is a team-based approach to care.**

1.True 2. False 3. I don’t know

**10. A goal of palliative care is to help people better understand their treatment options.**

1.True 2. False 3. I don’t know

**11. Palliative care encourages people to stop treatments aimed at curing their illness.**

1.True 2. False 3. I don’t know

**12. A goal of palliative care is to improve a person’s ability to participate in daily activities.**

1.True 2. False 3. I don’t know

**13. Palliative care helps the whole family cope with a serious illness.**

1.True 2. False 3. I don’t know
