# Supplementary material for: Physicians’ knowledge about palliative care in Bangladesh: A cross-sectional study using digital social media platforms
Source: PLoS One. 2021 Sep 1;16(9):e0256927. doi: 10.1371/journal.pone.0256927 (PMC8409647; doi:10.1371/journal.pone.0256927)
Supplement: Attachment — Submitted filename: Response to reviewers.docx [file pone.0256927.s003.docx]

**Physicians' knowledge about palliative Care in Bangladesh: A cross-sectional study using digital social media platforms**

**(Manuscript ID: PONE-D-21-09118)**

**Additional Editor’s comments to the authors-**

**Comment**: Please consider incorporating comments of reviewer 1 in the manuscript.

**Reply:** Thank you for your comments; we have revised the whole manuscript. We have considered and incorporated the revisions suggested by reviewer 1.

**Reviewer#1’s comments to the authors-**

**Comment 1:**  Abstract: Introduction: Last line: Better to replace the word ‘explore’ by ‘assess’. Same corrections needed at the end of background.

**Reply 1:** Thank you. The line has been revised and changed in the revised manuscript

**Changes in the text:**

- **Abstract : Introduction**

**Line 35**: **This study aims to assess the preliminary knowledge level and the misconceptions about this field among physicians.**

- **Background:**

**Line 112: This paper aims to assess the knowledge level and the misconceptions about palliative care among Bangladeshi physicians to understand the situation better and raise awareness.**

**Comment 2**: Better to mention the year of the study in the abstract too

**Reply 2:**  Thank you. The year of the study has been incorporated in the methods section of the abstract.

**Changes in the text:**

- **Abstract : Methods**

**Line 38-41**: **This cross-sectional study was conducted among 479 physicians using a self-administered structured questionnaire adapted from Palliative Care Knowledge Scale (PaCKs) on various digital social media platforms from December 2019 to February 2020.**

**Comment 3**: Table 3: Better to mention test statistics along with P value

**Reply 3:**  Thank you for pointing the issue. The test statistics is mentioned in the Table 3 of revised manuscript in the column titled “Test statistics”.

**Reviewer#2 comments to the author-**

**Comment 1**:  For decimal points, you used 1 decimal points. So it would be great to use 1 decimal point for response rate in line 45 and line 131; mean age in line 177 and table 1.

**Reply 1:** Thank you.The issue has been corrected in the revised manuscript body and also in Table 1.

**Changes in the text:**

- **Abstract: Results**

**Line 45: Almost equal number of physicians of both genders from four major specialties and their allied branches took part in the study (response rate 23.9%).**

- **Methods: Sample size and criteria:**

**Line 128-130: Initially, 2000 physicians who fulfilled the necessary inclusion criteria showed interest after learning about the study's objectives and purpose, but 479 returned with the complete response (response rate 23.9%).**

- **Results:**

**Line 176-177: Almost equal numbers of physicians from both genders took part in the study of mean age 28.9±3.6 years.**

- **Discussion:**

**Line 289-290: There is a chance of non-response bias as the response rate of this study is very low (23.9%).**

**Comment 2**: Typing errors in 156 and 159 (it should be "interquartile range"); line 163 and 223 (the tests must be "Fisher's exact test, and the Monte Carlo exact test")..

**Reply 2:**  Thank you. The typing error has been corrected in the line 156. Regarding the name of the tests performed in the line 162 and 223, we have conducted 3 tests. For the variables ‘Sex’ and ‘Qualifications’ we have performed ‘Chi square test’; the variable ‘Age’ we performed ‘Fisher’s exact test’; and for the variable ‘Specialties’ we performed ‘Monte Carlo exact test’. The name of all three tests has been also corrected and mentioned in the Table 3 of the revised manuscript.

**Changes in the text:**

- **Methods: Data analysis**

**Line 155-156: Knowledge level about palliative care was categorized into three categories according to the intraquartile ranges and median.**

**Line 162-164: Chi-square, Fisher's extract test, and the Monte Carlo extract test was done to compare the knowledge level with different study subjects' demographic variables setting the α level at 0.05.**

**Line 223: **Fisher’s Extract test; **Monte Carlo extract test; ***Chi square test; p value <0.05 considered as significant***

**Journal requirement comments to the authors-**

**Comment:** Please review your reference list to ensure that it is complete and correct. If you have cited papers that have been retracted, please include the rationale for doing so in the manuscript text, or remove these references and replace them with relevant current references. Any changes to the reference list should be mentioned in the rebuttal letter that accompanies your revised manuscript. If you need to cite a retracted article, indicate the article’s retracted status in the References list and also include a citation and full reference for the retraction notice.

**Reply:** Thank you. We have checked the reference list carefully and found the journal mentioned in the reference no 7 has been retracted. The retracted journal was “Merriman A. In the darkness of the shadow of death: a ray of hope: the story of Hospice Africa. J Palliat Care. 1993 Autumn;9(3):23-4. PMID: 8271102.”

So, we have replaced the above mentioned reference with an updated one. Both articles were conducted in almost same study setting and yielded the same conclusion. So we only updated the reference number 7 without any change in the text body.

**Changes in the text:**

- **Reference:**

**7. Grant L, Brown J, Leng M, Bettega N, Murray S. Palliative care making a difference in rural Uganda, Kenya and Malawi: three rapid evaluation field studies. BMC Palliat Care. 2011;10(1).**

**Dear Reviewers, we are grateful for your kind time and substantial review; we believe now the manuscript is more improved, which will satisfy you.**
